# Supplementary material for: Are carbon emissions trading and green financial instruments synergistic? -Comprehensive quantitative research based on content analysis
Source: PLoS One. 2024 Mar 7;19(3):e0298601. doi: 10.1371/journal.pone.0298601 (PMC10919601; doi:10.1371/journal.pone.0298601)
Supplement: S1 Data — (ZIP) [file pone.0298601.s001.zip › Data/地级市Z_F工作报告.zip!/╕ú╜¿/╒─╓▌╩╨/└┤╫╘╣1⁄2╓┌║┼í╛┬φ┐╦╩2╛▌═°í┐_2015.docx]

各位代表：

　　现在，我代表漳州市人民政府向大会作政府工作报告，请予审议，并请市政协各位委员和其他列席的同志提出意见。

　　一、2014年工作回顾

　　过去的一年，在省委、省政府和市委的领导下，在市人大及其常委会和市政协的监督支持下，我市各级政府认真贯彻党的十八大及十八届三中、四中全会和习近平总书记系列重要讲话精神，着力稳增长、调结构、促改革、惠民生，科学发展跨越发展取得新成效。全市生产总值2506.4亿元，增长11.3%；公共财政总收入263.8亿元，增长11%，地方公共财政收入169亿元，增长9.1%；规模工业增加值1087.4亿元，增长16.4%；全社会固定资产投资2134.8亿元，增长21.2%；外贸出口81.3亿美元，增长14.4%；实际利用外资10.1亿美元，增长7%；社会消费品零售总额785.5亿元，增长12%；居民消费价格总水平上涨2%；城镇居民人均可支配收入25741元，增长9.6%；农村居民人均可支配收入12690元，增长10.5%；城镇登记失业率2.02%；人口自然增长率9.25％。。三次产业结构由15.5∶48.8∶35.7优化为14.0∶49.8∶36.2。年度减排任务预计可以完成。

　　一年来的主要工作和成效是：

　　（一）经济增长稳中有进。面对经济下行压力加大的严峻形势，及时出台了支持龙头企业发展、加速工业跨越、稳定内外贸等一系列政策措施，强化要素保障，助推企业发展。更加注重发挥投资的关键作用，深入实施“项目建设年”和“大干100天”活动，全市347个在建重点项目完成投资953.4亿元，其中新开工153个，完成投资329.3亿元。一批交通、水利、电力等基础设施项目建成投用。更加注重发挥消费的基础作用，成功举办“漳州味·世界行”系列展销活动。积极发展网购等消费业态，加快建设电商平台，限额以上单位网上零售额增长329%。开展“智慧旅游年”活动，全年接待游客人数和旅游总收入分别增长19.3%和22.5%。更加注重发挥出口的拉动作用，加快出口退税步伐，推行关检合作“三个一”，提高通关贸易便利化水平，外贸出口增幅比全省高7.8个百分点。

　　（二）产业发展提速增效。产业转型升级步伐加快，9个重点产业产值3491亿元，增长19.4%。新增亿元企业29家，亿元以上规模工业总产值3476.2亿元，增长20.3%。古雷列入全国七大石化产业基地，万利达集团入选亚洲品牌500强。坚持创新驱动，完成技改投资451亿元，增长19.7%，新认定高新技术企业36家，高新技术产业产值850亿元，漳州高新区升级为国家级高新技术产业开发区。新增2个省级海洋产业示范园区。现代服务业加快发展，新增限额以上商贸流通企业88家。港口货物吞吐量5853万吨，增长5.9%。招银港区成为全国首批进境粮食指定口岸。全省首家邮储银行特色支行在东山成立，助农取款服务点实现行政村全覆盖。福能（漳州）融资租赁公司、海峡股权交易中心漳州运营中心正式开业，10家企业场外市场挂牌，敦信纸业、宏源表业在香港上市。本外币贷款余额1638.7亿元，增长15.4%；存款余额2096.6亿元，增长13.3%。特色现代农业迈出新步伐，粮食总产量保持稳定，我市获批国家现代农业示范区，新增国家级农业标准化示范区2个、国家级出口农产品质量安全示范区4个、设施农业2万亩。新登记农民专业合作社813家，新认证无公害、绿色和有机产品60个。“白芽奇兰”获中国驰名商标，“东山鲍鱼”获中国地理标志商标，“琯溪蜜柚”成为国家生态原产地保护产品。8个县（市、区）列入《赣闽粤原中央苏区振兴发展规划》，扶贫开发重点县、民族乡村加快发展，“造福工程”完成搬迁8050户，解决农村52.3万人的饮水安全问题。

　　（三）城乡面貌不断改观。城市总体规划、历史文化名城保护规划获批实施，启动建设闽南文化生态博览城核心区，基本建成25公里长的闽南文化生态产业走廊示范段。漳州古城被列为省重点文化产业园区。长泰成为全国美丽乡村标准化建设试点县。郊野公园龙文示范段项目、长泰上蔡村村庄整治项目获得2014年中国人居环境范例奖。新增2个国家历史文化名村、2个中国少数民族特色村寨、9个中国传统村落、1个全国生态文化村。入选全国文明城市，实施宜居环境建设行动计划，开展城区环境综合整治百日行动和农村建设“双百”行动，完成胜利路等主要城区道路立面景观改造，“数字城管”正式投入运营。“两违”治理累计拆违607万平方米；新改扩建城市道路238公里，新增供水管网221公里、雨污管网430.3公里、垃圾中转站32座、公交车209辆、公交线路20条，完成268公里河道清淤整治。

　　（四）重点改革实现突破。开展“深化改革年”活动，8个方面36项重点改革任务进展顺利。市县政府机构改革任务基本完成，事业单位分类改革稳步实施。推进工商注册制度便利化，新增各类市场主体4.7万户。推行行政审批规范化和服务标准化，率先在全省开展市级审批前置条件清理工作，加快“网上办事大厅”平台整合升级，保留行政审批事项88项，审批时限控制在法定时限60%以内。启动市属国有企业资产整合重组，年末资产总额923亿元。深化投融资体制改革，鼓励民间资本进入基础产业、社会事业以及特许经营领域，民间投资增长26.9%。扎实推进小城镇综合改革，全面放宽就业人员落户准入条件。继续深化农村综合改革，“三农”综合保险工作有序推进，村级组织运转经费有效保障，率先在全国开展林地占补平衡改革试点，农村集体土地所有权确权发证加快推进，流转耕地66.2万亩。

　　（五）对外开放稳步拓展。深化漳台交流合作，全年实际利用台资4.8亿美元，增长6.3%；对台出口额11.9亿美元，增长47.9%。在台湾设立“闽台商务股份公司”“漳州旅游服务中心”，台湾“中华两岸事务交流协会”入驻漳州，“海峡两岸新型农民交流培训基地”获批。成功举办农博会·花博会和海峡论坛漳州活动，成为第四批大陆居民赴台个人游试点城市，来漳台胞超过14.8万人次。提高招商引资实效，全年对接“三维”项目563个，总投资7776亿元，新引进世界500强企业2家，新批千万美元以上项目52个。积极融入“21世纪海上丝绸之路”建设，与东盟贸易额15.1亿美元，增长8.4%；实际利用东盟资金3637万美元，增长1倍。举办全国“海丝”文化遗产精品九城市联展和“闽南风·漳州味”文化周活动。外事侨务工作积极有效。厦漳泉同城化第一、二批项目有序推进。闽粤经济合作区规划建设步伐加快。援藏援疆援宁工作持续推进。

　　（六）生态建设取得实效。以铁的决心、铁的措施、铁的纪律开展水环境整治，九龙江流域漳州段Ⅲ类水质达标率达93.6%，县级以上集中式饮用水源水质达标率99.3%。落实节能减排责任制，抓好十大重点领域工程节能改造。完成市区PM2.5监测及信息发布，空气质量优良率平均99.7%。加强“三沿一环”治理、“五海”保护和“六大工程”建设。扎实推进“四绿”工程、“六大森林工程”建设，完成造林绿化19万亩。治理水土流失面积27.3万亩。生态市创建通过省级考核验收，长泰、南靖获评福建首批“国家生态县”，东山通过“国家生态县”考核验收，43个乡镇被命名为国家级生态乡镇。我市被命名为“中国温泉之城”。

　　（七）群众生活持续改善。为民办实事项目全面完成。新开工建设中小学扩容工程5.8万平方米，新增公办幼儿园16所，全市义务教育学校全部完成标准化建设。33个卫生重点项目顺利推进，新增医院病床位2139张、卫技人员1402名。龙海成为全国县级公立医院综合改革试点县。成功举办第十五届省运会、第九届老健会和第七届市农运会，新改扩建5个大型场馆和23个中小型场馆。博物馆、艺术馆、规划展示馆开工建设。诏安荣获“中国长寿之乡”称号。芗剧《保婴记》、小说《援疆干部》获“五个一工程”奖，《绿色的青春》荣获“十大纪录片”奖。“三平祖师信俗”列入国家级非物质文化遗产名录。新增城镇就业5.1万人、转移农村劳动力7.1万人。城乡居民养老保险实现一体化。全市新农合参合率达到99%以上，城镇居民基本医疗保险政府补助标准年人均提高到320元，城乡居民大病保险全面实施。农村低保标准年人均提高到2100元，企业退休人员月人均养老金提高到1902元，退休村、居主干实现同等待遇。新开工建设保障性安居工程12970套，基本建成15876套。完成首批农村特困家庭危房改造。深入推进“平安漳州”建设，加强社会治安综合治理。全面推行和谐征迁，信访积案有效化解。扎实开展安全生产专项整治，有效保障食品药品安全。推进军民深度融合，军政军民团结巩固发展。妇女儿童、青少年、民族宗教、新闻出版、社会科学、地方志、档案、气象、防灾减灾、残疾人、红十字会、工会、人防、物价等各项工作都取得新的进步。

　　（八）作风建设不断加强。党的群众路线教育实践活动和“作风建设年”活动取得阶段性成果，全面停止新建楼堂馆所，完成超标办公用房清理工作，“三公”经费支出下降29.4%。切实改进文风会风，以市政府名义召开的会议减少15.6%，下发的文件减少3.4%，市级考核检查项目减少67.3%。自觉接受人大及其常委会、政协监督和社会舆论监督，全年共办理市人大代表建议175件、市政协提案316件，办结率100%。政府与政协、法院、工会互动联系机制不断完善。实施政府信息公开办法，完善绩效评估，行政问责、行政监察和审计监督力度加大，廉政建设和反腐败工作深入开展。

　　成绩的取得来之不易，这是省委、省政府和市委正确领导的结果，是全市人民齐心协力、奋力拼搏和各方面大力支持的结果。在此，我代表市人民政府向全市人民，向人大代表、政协委员、各民主党派、工商联、各人民团体、无党派人士、离退休老同志和社会各界人士，向中央、省驻漳机构和驻漳部队、武警官兵、公安政法干警，向关心支持漳州发展的港澳台同胞、海外侨胞和国际友人，表示崇高的敬意和诚挚的感谢！

　　在总结成绩的同时，我们也清醒地认识到发展还面临不少困难和问题，主要是：经济下行压力加大，重大项目储备不足，企业生产经营困难增多；龙头企业不多、带动力不强，新兴产业和服务业规模偏小，产业整体竞争力偏弱；节能减排形势严峻，水、大气、土壤等环境保护压力加大；城乡基础设施配套不足，规划建设和管理水平有待提升；教育、医疗、文化等社会事业欠账较多，资源不足与分配不均并存；食品安全、安全生产、社会治安等公共安全领域还存在不少隐患；政府职能转变不到位，干部队伍庸懒散、不作为、慢作为、乱作为现象仍然存在。这些问题，我们将高度重视，认真解决。

　　二、做好2015年工作

　　我们要按照中央和省委、省政府对经济发展新常态提出的新要求，全面贯彻落实市委的重大决策部署，凝心聚力，乘势而上，全力做好“十二五”收官之年工作，科学编制“十三五”规划，为实现漳州发展新目标迈出坚实步伐。

　　政府工作的总体要求是：全面贯彻党的十八大、十八届三中、四中全会精神，深入学习贯彻习近平总书记系列重要讲话和来闽考察重要讲话精神，认真落实中央支持福建进一步加快经济社会发展的一系列政策措施，按照市委十届八次、九次全会部署，主动适应经济发展新常态，坚持稳中求进，突出好中求快，持续开展“项目建设年”“深化改革年”“作风建设年”活动，加速工业跨越，提升现代农业，做大第三产业，深化改革开放，注重生态保护，加强民生保障，全力推进漳州科学发展跨越发展，在新的起点上加快建设富美漳州，为建设机制活、产业优、百姓富、生态美的新福建做出更大贡献。

　　今年经济社会发展的主要预期目标是：全市生产总值增长11.5%左右；公共财政总收入增长10%左右，地方公共财政收入增长6%左右；全社会固定资产投资增长22%左右；规模工业增加值增长16.2%左右；外贸出口增长8%，实际利用外资增长6%；城镇登记失业率控制在3.6%以内；社会消费品零售总额增长13%左右；居民消费价格总水平涨幅控制在3%左右；人口自然增长率控制在9.5％。以内；城镇居民、农村居民人均可支配收入均增长11.5%；完成单位生产总值能耗、化学需氧量、二氧化硫、二氧化碳、氨氮、氮氧化物等节能减排任务。

　　（一）全力落实科学发展跨越发展行动计划

　　打造四大经济增长极。推动四大经济增长极真正成为带动全市经济发展的强大引擎和重要支撑。一是古雷港经济开发区。积极申报设立古雷综合保税区,重点抓好中石化古雷炼化一体化等12个生产性项目、古雷作业区8号泊位等22个基础设施项目的开工建设，全面启动古雷半岛东西两侧填海造地工程，国庆节前基本完成居民整体搬迁任务、年底实现全线封关。二是厦门港南岸新城。加快核心区概念性城市设计，积极推进厦漳同城大道、厦漳城际轨道R3线、沿海大通道、后石港区3号泊位等基础设施项目和中海油LNG接收站、正新橡胶技改扩建、福欣不锈钢冷轧、特殊钢深加工产业园、纸品包装产业园等产业项目，加快角美城市副中心建设，积极构建新兴生态型港口工贸城市。三是漳州高新技术产业开发区。加快编制高新区扩区总体规划，重点推进南江滨路、圆山大道、片仔癀生物医药产业园、“五馆一歌”等先行启动项目，加快中心城区跨江南扩。四是环东山岛经济区域。加快区域协同发展规划编制，重点推进东山生态环岛公路等基础设施项目建设，做大做强东山玻璃及新材料产业园、海洋生物科技产业园，诏安海洋生物产业园、云霄光电产业园、常山旅游华侨城，规划建设闽粤经济合作区。

　　加大重点领域投资。按照“两个50%以上”的要求，力争全社会固定资产投资完成2600亿元。工业领域计划投资1000亿元，重点推动腾龙芳烃PX改扩建、锦江石化芳烃-己内酰胺一体化、台湾中石化烯烃芳烃一体化等项目开工建设。能源领域计划投资76亿元，力争漳州核电项目开工建设、古雷华能热电项目年底动工，加快500千伏东林变电站等项目建设。城建领域计划投资800亿元，实施城乡基础设施“六项提升工程”，重点推动东墩污水处理厂、城市棚户区改造等项目。交通领域计划投资175亿元，加快建设厦漳同城大道、沿海大通道，开工建设漳梅高速云霄至平和段、国省干线联六线、联十一线、漳江湾特大桥等项目，确保漳永高速、东山联络线建成通车。服务业领域计划投资210亿元，重点推动大乌山旅游综合开发、漳龙物流园区、盛辉物流、海峡农产品物流城二期、漳州木材交易中心等项目建设。生态领域计划投资65亿元，突出抓好闽南文化生态产业走廊、九十九湾生态绿道、八尺门退堤还海、南北港海洋生态修复、城洲岛生态修复等项目。农林水领域计划投资100亿元，重点推进枋洋水利枢纽工程、东山岛外引水第二水源工程、九龙江防洪排涝工程漳州段等水利项目。社会事业领域计划投资100亿元，重点推动闽南文化生态博览城、市社会福利中心等项目。

　　强化要素支撑保障。实行“一月一督查、半月一调度”，完善协调、考核、激励机制，开通审批绿色通道，落实项目用地、用林、用海、减排总量、火工油品等要素需求。完善政银企对接长效机制，推广规范PPP等建设运营模式，努力破解资金瓶颈。推进和谐征迁，变安置为安居。全面推行标准化管理，打造阳光工程、优质工程。

　　（二）坚定不移加速工业跨越

　　培育壮大龙头企业。全面落实产业龙头促进计划，实行“一企一策”，扶持50家市级以上重点龙头企业，力争培育4家以上主营业务收入超百亿元企业。加快建设石油化工、食品加工、特殊钢铁、装备制造四大基地，加大古雷石化中下游产业链招商力度；加紧推进福欣40万吨节镍不锈钢项目、100万吨不锈钢冷轧项目和特殊钢深加工产业园建设，加速形成高端不锈钢深加工产业集群；大力发展南靖、华安数控机床装备制造和漳州开发区海洋工程装备制造产业；加快百威英博啤酒扩建、泡吧食品、盈丰食品工业园等项目建设。积极引办一批高端化、高质化、高新化临港工业项目。争取钟表产业园早日落户。

　　促进工业扩量提质。落实加速工业跨越发展实施意见等政策，健全以税收贡献、创新转型为导向的产业促进机制。加大对上市或上市后备企业扶持力度，鼓励企业增资扩产、建设总部，力争新增上市企业3家、场外市场挂牌15家。强化工业运行调度，突出抓好融资服务，完善企业资金应急处置机制。落实甲供甲控，优先选购本地名优产品。鼓励企业参与市外招投标、扩大网上销售，促进线上线下融合发展。支持企业构建产业联盟。

　　大力推进科技创新。加快食品科技研究院、工业设计创新公共服务平台、创新创意中心建设。实施新一轮企业技术改造，抓好200个市级以上技改项目建设，完成技改投资450亿元以上。落实固定资产加速折旧、研发费用加计扣除等鼓励创新政策，支持企业主导或参与国际、国家和行业标准修订。发挥“6·18”“蓝火计划”等成果对接平台作用，实施“人才生根”战略，开展“两院”技术成果推介对接专场，规划建设“院士小镇”。实施“数控一代”机械产品创新应用示范工程。实施专利运用行动计划。出台扶持措施，加快互联网经济、物联网等新业态发展。

　　（三）发展壮大特色现代农业

　　强化粮食安全保障。全面落实惠粮政策，抓好10个万亩粮食高产示范片、1个省级粮食产能区和2个再生稻示范县项目建设，完成粮食生产目标。继续实施“引粮入漳”工程，加快建设6个现代化粮食储备库，支持规模化粮食生产流通企业发展。加强以水利为重点的农村基础设施建设，推进高标准农田建设，继续抓好土地整理、抛荒山垅田复垦改造。

　　提升农业品种、品质、品牌。突出培育良种、提升品质、创建品牌，建设一批全省一流的示范基地。出台现代种业扶持政策，整合做大种子龙头企业。推进农业设施化规模化标准化，加快4个省级农业标准化示范区建设，新建3个千亩以上、3个500亩以上设施农业温室大棚基地。扶持远洋渔业发展，建设5个渔港和10艘远洋渔船，推进东山远洋渔业码头建设。大力发展农产品加工业，培育农业龙头企业，力争实现年销售收入10亿元以上龙头企业20家、超50亿元1家。推进水仙花保护基地建设，打响白芽奇兰品牌。大力发展“三品一标”，加强农产品质量安全可追溯平台建设。抓好动植物重大疫病防治。加强气象现代化建设，提高防灾减灾能力。

　　创新农业经营方式。加快农村土地承包经营权和水域滩涂养殖确权登记颁证工作，放活土地经营权，实现适度规模经营。扶持发展家庭农场，规范提升农民专业合作社，大力发展经营性服务组织，完善与农户的利益联结机制。继续实施新型职业农民培育工程，依托“海峡两岸新型农民交流培训基地”，加大农民技能培训。建立财政扶持农业保险专项基金，加快扶贫开发重点县支农信贷协会和村级融资担保基金试点创建工作。

　　（四）更加重视发展服务业

　　加强规划引导和政策扶持。编制城市服务业发展规划，进一步优化布局、丰富业态，形成若干个层次清晰、各具特色的现代服务业集聚区。强化政策扶持，出台加快服务业发展政策措施。稳步推进“退二进三”，鼓励利用存量土地、旧厂房旧仓库发展现代服务业。支持龙文推进省级服务业综合改革试点。

　　促进生产性服务业与制造业有机融合。一是强化金融对实体经济的服务。新增信贷规模260亿元以上。支持发展民营银行等中小金融机构，鼓励各金融机构设立专业支行或特色银行，推进村镇银行实现县域全覆盖。突破资产证券化，支持本地企业集团设立财务公司，鼓励融资租赁、股权交易、产业基金入漳搭建平台，开展经营活动。加强金融生态建设，完善征信体系，防范金融风险。二是发展壮大现代物流业。加强物流节点、园区和配送中心建设，完善仓储、冷链物流设施，引进培育大型物流企业，加快推进7个物流项目建设。三是扶持拓展信息服务业。扶持电子商务发展，加快四大电商平台和一批电商园区建设，吸引第三方电商企业和区域快递总部入驻，促进电商与物流快递协同发展；深化“数字漳州”建设，完善提升市级电子政务公共平台，推进大数据开放开发，建设智慧城市。支持发展创意咨询、商务会展、检验检测等服务业。

　　提升生活性服务业质量效益。一是加快发展旅游业。突出闽南文化、美食文化、“海丝”文化特色，吸引更多游客来漳州登山、观海、戏水、看楼、泡温泉、赏名花、尝鲜果，推动观光游向休闲度假游转变。启动大乌山景区规划建设。实施景区提升和旅游线路建设工程，完善吃住行游购娱配套，抓好景区餐饮卫生整治，打响漳州旅游品牌。二是促进房地产市场健康发展。完善扶持政策，支持群众首套房、首改房等刚性住房需求，激发市场活力。三是做大做强文化产业。加快龙人古琴文化村、牛庄文创园等项目建设，支持发展文化创意、设计服务等产业。四是繁荣商贸市场。扶持“限上企业”发展，新增“限上企业”90家以上，改造提升传统流通业态，完善农村流通网，促进城乡流通换挡升级。五是大力发展健康服务业。支持社会资本投资健康服务业，鼓励发展家政、社区、养老等服务业。

　　（五）积极稳妥推进新型城镇化

　　优化城镇布局。坚持“田园都市、生态之城”定位，实施新型城镇化规划，中心城市城镇组群以漳州高新区、厦门港南岸新城等区域为主要载体，推动“中心东移、跨江南扩、面海拓展”战略加快实施；环东山岛城镇组群突出科学规划引领，进一步做大做美县城和中心城镇，做强做优特色产业。协同推进厦漳泉大都市区同城化。推进县（市）域城乡总体规划试点，推行“多规合一”，建立规划管理审批“一张图”系统。

　　拓展中心城区。积极推进“东西南北中”五大景观区域建设，加快建设闽南文化生态博览城和闽南文化生态产业走廊，完善提升郊野公园建设与管理。加快城市道路桥梁和公共服务设施建设，完成新一批“五千工程”，抓好城区与县域通道建设。发展城市公共交通，优化公交路网，加快公交站场和停车场建设，缓解“行车难”“停车难”问题。推动城市地下管网综合管廊建设。注重保护“五古”文化资源，加快古城保护性开发，彰显闽南古风。

　　做强县域经济。按照生态优先、产城融合的理念，进一步优化布局、完善配套、集约用地、突出特色，做强“一县一园区”。深化山海协作，鼓励共建产业园区。按照次中心城市的标准，加强县城规划建设，鼓励实施道路“白改黑”、缆线“上改下”、雨污“合改分”，形成功能完善、各具特色的城镇风貌。推行无门槛居住证制度和差别化落户政策，扩大基本公共服务覆盖面，加快公共租赁住房建设，推动农业转移人口就近就地市民化。开展“富美乡村”规划编制试点和“双百”行动，建设一批城市人向往、农村人留恋的富美示范村。

　　营造宜居环境。巩固创建全国文明城市成果，推广大城管试点经验，开展无物业小区整治，强化城市精细化管理，提升城市建设管理水平。继续实施宜居环境建设计划，保持“两违”治理高压态势，延伸拓展“点线面”攻坚，提升“三边三节点”整治水平，突出抓好县市区交界处环境整治。实施农村生活垃圾三年专项治理行动。提升城市绿化美化水平。加强城市噪音污染治理，推进移风易俗，倡导文明生活方式。

　　（六）持续深化改革开放

　　加快重点领域和关键环节改革。加强自贸区政策研究，主动接受福建自贸区辐射，借鉴先进经验，力争有所作为。继续简政放权，精简前置审批，规范中介服务行为，全面推行行政服务标准化，加快建设“四清单、一张网”。下放环东山岛经济开发区产业项目市级审批权限。稳步推进事业单位分类改革，深化商事制度改革。整合规范政府采购、工程招投标等公共资源交易平台，开展公务用车制度改革，健全政府购买公共服务机制。推进全口径预算管理制度改革，加快国企整合重组，鼓励社会资本投资公共事业领域。

　　打造“海丝重要节点城市和先行示范区”。围绕构建“三大示范区”，以项目为载体，以产业为重点，全面融入福建省21世纪海上丝绸之路核心区建设。深化与中东、东盟等地区在能源供应、海洋船舶、生物医药、新材料等领域的合作。加强与海外侨团联系，开展侨资回归工程，创建福建-东盟经贸合作试验区。修编《漳州华侨史》，做好华侨华人族谱对接。加快“海丝”申遗步伐，深入挖掘漳州月港史料遗存，加强“海丝”遗址修复与保护。传承漳绒、漳缎、漳瓷等“海丝”文化遗产，建设漳州“海丝”文化精品展示厅。

　　加大招商引资力度。突出招大商、选好商，大力推行小分队招商、龙头企业招商、专项招商、靶向招商，努力实现引进世界500强、全球行业龙头企业、全国百强民企新突破。加快实施“回归创业工程”，举办漳州海商创业大会。整合招商机构，组建专门招商队伍，强化招商绩效考核，制定各级商会、行业协会、社团等参与招商的奖励办法。突出经贸主题，坚持专业化、市场化、特色化方向，务实高效办好各类展会。

　　提升外贸竞争力。主动参与厦门东南国际航运中心建设，推动厦门港“同港同策”和“关检协作”，优化口岸通关环境，吸引货源回流、企业回归，做大口岸经济。培育外贸龙头企业，实施山区出口企业差别化政策，支持传统优势出口行业、重点出口基地转型升级。办好“漳州味·世界行”系列活动，鼓励企业开展境外收购、设立国际营销网店，提高市场占有率。

　　（七）积极推动漳台深度融合

　　深化产业对接合作。以石化、食品、电子、特殊钢、装备制造等产业为重点，实施产业对接升级计划，加快建设漳州深化两岸产业合作示范区。多跑台湾，洽谈合作，对接项目，以台引台，大力引进台湾百大企业、行业龙头和科技型中小企业。以共建平台、共创品牌、共拓市场为重点，密切与台湾行业协会常态化联系，促进台资企业转型升级和漳台双向投资，两岸联手赚世界的钱。

　　促进人文交流往来。积极引进发展台湾文化创意产业，加快建设一批对台文化交流合作基地，开工建设星云书院、宝岛时代村等项目。发挥祖地文化优势，加强涉台文物保护，深化族谱对接，加快陈元光漳台文化公园建设，扩大开漳圣王文化等漳台民间信仰、民俗文化的宣传和交流。深化乡镇对接，持续开展同名村联谊和宗亲交流，促进乡情延续。

　　（八）努力争创全国生态文明先行示范区

　　强化生态环境建设。认真落实全国生态文明先行示范区《实施方案》，积极争创国家森林城市。统筹推进“六大工程”，加大“五海”资源、红树林、基本农田和矿产资源保护。深入实施“四绿”工程，重点推进沿海防护林断带合围，完成造林绿化16.69万亩。加强水土流失治理，实施山体、水域、海岸带生态修复工程以及工矿废弃地恢复治理，巩固“三沿一环”青山挂白治理成果。严格执行耕地“先补后占、占一补一”、林地“先入库后出库”原则，推进林地占补平衡改革试点工作。推动实施企业环境污染责任保险，落实重点流域生态补偿办法。

　　抓好水、大气、土壤污染治理。持续开展“三铁三级三年行动”，落实河长责任制，深入推进禽畜养殖污染治理，加强饮用水源地保护，继续加大内河整治力度，加快内林、三湘江引水泵站以及污水管网建设，促进水质持续好转。抓好城区胶合板企业污染清理整治，落实大气污染防治行动计划实施细则，持续提高空气质量。启动土壤污染治理与修复工程试点，强化重金属和持久性有机污染物污染防治，推广使用高效、低毒、低残留农药，降低土壤污染风险。深入实施环境监管能力建设三年行动，强化环境风险预警和防控。

　　完成节能减排目标。严格落实新《环保法》和环保监管“一岗双责”，抓好重点减排工程建设，确保完成东墩污水处理厂等5个国家减排责任书项目，加快实施27个污水处理厂新改扩建工程，新建污水配套管网273公里，提高城镇污水收集处理率。全面实施能耗强度、碳排放强度和能源消费总量控制，落实重点行业脱硫脱硝工作，加快淘汰落后产能。严格节能评估审查和环境影响评价，严控高耗能、高排放项目。

　　（九）切实保障和改善民生

　　增强社会保障能力。办好18件为民办实事项目。落实就业创业扶持政策，加大职业技能培训，新增城镇就业5万人，转移农村劳动力6.8万人。提高企业退休职工和城乡居民基础养老金，实施机关事业单位养老保险制度改革。完善征迁补偿机制，妥善解决失地农民就业和社会保障问题。积极推动“三保合一”，增强城乡居民、城镇职工大病保障能力，推动城乡低保、农村“五保”扩面提标。加大危旧房和棚户区改造，完善社区配套，新开工建设保障性安居工程8590套，基本建成7000套。大力发展老年服务事业和产业，支持发展红十字、社会福利、慈善和残疾人事业，保障妇女儿童、青少年合法权益。

　　实施科学扶贫精准扶贫。用好用足各类扶持政策，健全专项扶贫、对口扶贫、社会扶贫机制，落实市县两级扶贫开发基金，重点抓好3个省级扶贫开发重点县、17个欠发达乡镇、183个贫困村扶贫开发。强化领导联系、山海协作、对口帮扶。加快推进“造福工程”，完成6000户以上搬迁任务。

　　办好教育文化事业。推进教育资源向农村地区、贫困地区、城市新区倾斜，开工建设中小学扩容工程4万平方米，启动农村义务教育薄弱校改造计划，完善校安工程配套。扩大普惠性学前教育资源，新改扩建公办幼儿园3.2万平方米。发展职业教育，规范民办教育，扶持特殊教育，重视老年教育。加快推进市县两级重点文化设施、乡村文化健身场所建设，鼓励社会资本参与公共文化服务体系建设。加强历史文化名城、名镇、名村、名街保护，保护好非物质文化遗产和传统艺术，创作一批富有漳州特色的艺术精品。做好第二轮志书和年鉴的编纂工作。建成市数字档案馆，加强县级综合档案馆建设。

　　推进医改纵深发展。继续开展“医疗卫生事业建设三年行动”，新增床位1531张、卫技人员1000名，培训全科医生和乡村医生4000名。全面推进公立医院综合改革，完善药品集中采购和配送制度，加强基层医疗卫生机构建设，推动形成分级诊疗制度。提高人均基本公共卫生服务经费标准，扩大服务覆盖面。鼓励社会资本办医，力争民营医院病床位总数占全市的20%。提高出生人口素质，促进人口长期均衡发展。

　　确保社会和谐稳定。深化“平安漳州”建设，推进城乡社区网格化服务管理，创新立体化社会治安防控体系，依法打击违法犯罪活动。加强矛盾纠纷和信访积案排查化解，按照“路线图”和“七项机制”处理信访事项。严格落实安全生产“党政同责、一岗双责”，深入开展道路交通、火灾隐患、重点行业、打非治违等专项整治，推进企业安全生产标准化建设提升工程三年行动。强化食品药品安全监管，实施“食品放心工程”三年行动。做好村（居）换届选举工作，提高村（居）两委待遇水平。加强行政复议规范化建设。积极开展普法宣传，做好法律服务和援助。扎实推进国防动员、外事侨务、民族宗教工作。

　　各位代表！人民政府始终要把人民的利益放在首位，全心全意为人民服务，加快建设服务型政府和法治政府。

　　依法行政，彰显公信力。政府任何时候都必须坚持在党的领导下、在法治轨道上开展工作，依法履职，遵循程序，明确权力和责任清单，做到“法无授权不可为”“法定责任必须为”。健全依法决策机制，深化行政执法体制改革，规范行政执法行为，完善规范性文件的报备制度。依法接受人大监督、民主监督、司法监督、社会监督和舆论监督。全面推进政务公开，让权力在阳光下规范运行。

　　优化服务，激发新活力。进一步减政、减负、减支,以政府权力的“减法”换取市场活力的“加法”,以政府的工作效率换取企业的发展效益,以干部的辛苦指数换取群众的幸福指数。倡导“马上就办”，破除“熟人经济”，狠抓效能建设，强化绩效管理，整治不作为、乱作为、慢作为。最大限度减少审批、优化流程，让企业和群众办事更方便、更快捷、成本更低。

　　转变作风，提高执行力。践行“三严三实”，坚持脚踩地上、眼睛向下、心装群众，带着感情、带着责任多关注群众的“票子、房子、孩子、身子和菜篮子”。坚持一切从实际出发，崇尚实干，狠抓落实，对看准、议定的事，立说立行，责任到人，干一件，成一件。

　　廉洁从政，增强免疫力。坚持厉行节约，反对铺张浪费，确保“三公”经费只减不增，群众过好日子，政府过紧日子。落实党风廉政建设责任制，推进源头治理，强化土地出让、工程建设、政府采购等重点领域的监管，让公务人员心有所畏、言有所戒、行有所止。牢记“教训就在身边、诱惑就在眼前、陷阱就在脚下、成败就在手中”，不收不送，廉洁从政。

　　各位代表！做好今年工作，任务艰巨，责任重大。让我们紧密团结在以习近平同志为总书记的党中央周围，在省委、省政府和市委的领导下，紧紧依靠全市人民，抢抓机遇，锐意进取，扎实工作，为建设富美漳州而努力奋斗!
